# EXPANDING INSIGHTS INTO LATE-LIFE DISABILITY USING OBJECTIVE ASSESSMENTS IN NHATS

**DOI:** 10.1093/geroni/igad104.0541

**Published:** 2023-12-21

**Authors:** Jennifer Schrack, Elena Fazio

## Abstract

The National Health and Aging Trends Study (NHATS) is a NIA-funded national panel study that conducts annual in-home interviews with a representative sample of US Medicare beneficiaries ages 65 years and older. Designed as a platform for the scientific study of late life disability trends and trajectories, NHATS fosters research to guide efforts to maximize health, promote independent functioning, and enhance quality of life at older ages. NHATS has collected performance-based measures of physical function since its inception (2011). In 2021 NHATS responded to expanded interest in objective measures of sensory loss and physical activity, by adding measures of vision (visual acuity, contrast sensitivity), hearing (pure tone audiometry), and physical activity (accelerometry). This symposium will introduce these measures and demonstrate their scientific application to answer key research questions related to physical and cognitive health. Dr. Schrack will present an overview of the accelerometry data collection procedures, as well as results by key demographic and health-related features. Dr. Twardzik will examine the association between features of participants’ social and physical environments and patterns of accelerometry-based physical activity. Dr. Martinez-Amezcua will examine the association between hearing and phenotypic frailty. Dr. Davoudi will present evidence on the links among sensory and motor function and risk of dementia. Finally, Dr. Wanigatunga will describe the association between measures of hand and leg strength and dementia incidence. Collectively, these presentations will highlight population-level associations of objective measures of health and function with physical and cognitive outcomes to advance the science of disability in older adults.

